# Intraoperative Evaluation of Bladder Perfusion Using Indocyanine Green Fluorescence Imaging During Total Pelvic Exenteration After Interruption of Blood Flow From the Internal Iliac Vessels

**DOI:** 10.1002/ags3.70131

**Published:** 2025-11-24

**Authors:** Mamoru Uemura, Chikako Kusunoki, Mao Osaki, Hiroshi Kusafuka, Satoshi Higuchi, Yuki Sekido, Mitsunobu Takeda, Tsuyoshi Hata, Atsushi Hamabe, Takayuki Ogino, Norikatsu Miyoshi, Koji Munakata, Hirofumi Ota, Yuichiro Doki, Hidetoshi Eguchi

**Affiliations:** ^1^ Department of Gastroenterological Surgery, Graduate School of Medicine The University of Osaka Suita Japan; ^2^ Department of Gastroenterological Surgery Ikeda City Hospital Ikeda Japan

**Keywords:** bladder perfusion, Indocyanine green fluorescence imaging, internal iliac vessels, lateral lymph node dissection, rectal cancer

## Abstract

**Background:**

In lateral lymph node dissection (LLND) for locally advanced or recurrent rectal cancer, concomitant resection of the internal iliac vessels is sometimes required. Because the bladder receives its primary blood supply from branches of the internal iliac artery, concerns arise regarding bladder perfusion when these vessels are resected. However, the extent to which bladder perfusion depends on the internal iliac system remains unclear.

**Methods:**

Between 2020 and 2023, 25 patients with locally advanced or recurrent rectal cancer who underwent total pelvic exenteration (TPE) with bilateral LLND were prospectively enrolled. After division of all ventral branches of the internal iliac vessels, including the umbilical, obturator, and vesical vessels, bladder perfusion was evaluated intraoperatively using indocyanine green (ICG) fluorescence imaging. Time to visualization of bladder perfusion was recorded.

**Results:**

Bladder perfusion was successfully visualized in all patients without complications. Median time to visualization was 30 s (interquartile range, 20–50). Perfusion originated exclusively from the pubic side, and fluorescence of the entire bladder wall was confirmed after mobilization. Patients were classified into early (≤ 30 s) and delayed (> 30 s) visualization groups. Older age was significantly associated with delayed visualization (*p* = 0.044), whereas no other clinical or surgical factors showed associations.

**Conclusions:**

Bladder perfusion was maintained even after complete interruption of both arterial supply and venous drainage from the internal iliac vessels, owing to blood supply from the pubic side. These findings support the safety of internal iliac vessel resection during extended LLND in rectal cancer surgery.

## Graphical abstract

Intraoperative ICG fluorescence imaging demonstrated that bladder perfusion was maintained through blood flow from the pubic side, even after complete division of the internal iliac vessels during total pelvic exenteration.

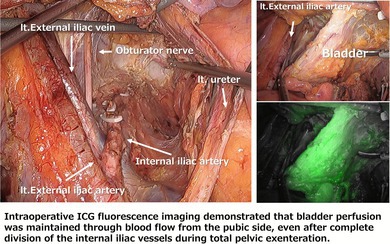

## 1 Introduction

Lateral lymph node dissection (LLND) is required in selected cases of surgery for patients with locally advanced rectal cancer [1, 2], and in some cases, combined resection of the internal iliac vessels is performed to ensure complete removal of metastatic nodes [3]. Since the primary blood supply to the bladder arises from branches of the internal iliac artery, the impact on bladder perfusion becomes a major concern when these vessels are resected. However, there is currently limited evidence on how much preservation of these branches is necessary to maintain adequate bladder perfusion.

The usefulness of indocyanine green (ICG) fluorescence imaging for assessing tissue perfusion has been widely reported in recent years. For example, several studies have demonstrated that ICG fluorescence angiography is a safe and feasible tool for the intraoperative assessment of tissue perfusion during colonic resection [4, 5, 6]. Furthermore, a recent multicenter randomized clinical trial showed that intraoperative perfusion assessment using ICG fluorescence imaging significantly reduced the incidence of anastomotic leakage in rectal cancer surgery, providing further evidence of its clinical utility [7]. Understanding whether a substantial blood supply exists from the pubic side is crucial for ensuring that bladder perfusion can be preserved when performing combined resection of the internal iliac vessels.

A previous report evaluated bladder perfusion in 23 patients with rectal cancer by temporarily clamping the internal iliac artery during surgery and measuring tissue perfusion using a Doppler tissue blood flowmeter. Although a transient decrease in bladder perfusion was observed, no ischemic damage leading to necrosis was reported. Interestingly, the reduction in bladder perfusion was more pronounced when the artery was clamped distal to the superior gluteal artery, compared to clamping proximally at its origin from the common iliac artery [8]. This suggests that more proximal clamping may preserve collateral pathways via the obturator, internal pudendal, and inferior vesical arteries, allowing retrograde perfusion from the external iliac artery and trunk vessels. However, this study only assessed the effects of arterial clamping and did not include anatomical dissection of the vessels. In particular, since multiple small branches are often present around the inferior vesical vessels, accurate evaluation of perfusion changes may require clear exposure of these vessels. Moreover, the study did not involve extended procedures such as LLND or en bloc resection of the internal iliac vessels, and therefore did not assess the impact of such anatomical interventions on bladder perfusion. Perfusion changes should be interpreted carefully, as they can be significantly affected by both the location of the vascular branches and the degree of interruption.

Another report described outcomes in seven patients with bladder cancer who suffered from severe and intractable hematuria and were not candidates for curative surgery due to poor general condition or advanced disease stage. Bilateral embolization of the internal iliac arteries was performed for hemostasis, and despite the complete interruption of blood flow through both the superior and inferior vesical arteries, no serious complications, such as bladder necrosis, were observed [9]. However, even in this study, anatomical dissection to fully expose and occlude all branches supplying the bladder was not performed, and thus, the findings should be interpreted with caution to avoid overestimation.

It has been suggested that bladder perfusion is not solely dependent on branches from the internal iliac vessels, but is also supported by blood supply from the pelvic sidewall. However, there have been no reports in which real‐time intraoperative bladder perfusion was evaluated under conditions where the feeding vessels were actually ligated and divided. This study aimed to determine whether perfusion from the pelvic sidewall to the bladder is adequately maintained under such conditions. Specifically, during total pelvic exenteration (TPE), we intraoperatively assessed bladder perfusion using ICG fluorescence imaging after complete interruption of the internal iliac vessels and their branches, including ligation and division of the umbilical vessels and obturator vessels. Such an evaluation of bladder perfusion can only be performed in cases where the bladder is resected.

## 2 Materials and Methods

### 2.1 Patients

Between 2020 and 2023, patients with locally advanced or recurrent rectal cancer who underwent pelvic exenteration with concomitant bladder resection, corresponding to TPE, at The University of Osaka Hospital and Ikeda City Hospital were prospectively enrolled. All patients were assessed preoperatively to confirm the absence of iodine allergy. A total of 25 patients were included, and intraoperative bladder perfusion was evaluated. The clinical characteristics of these patients are summarized in Table 1.

**TABLE 1:** Patient characteristics and surgical procedures.

Characteristic	Value (*n* = 25)
Age, years, median (IQR)	70 (61–72)
Sex	
Male/female	19/6
ASA physical status	
I/II/III	1/22/2
Diabetes mellitus	
No/yes	22/3
Smoking status	
Nonsmoker/ex‐smoker/current smoker	13/9/3
Body mass index, median (IQR)	20.15 (19.2–22.2)
Diagnosis	
Primary rectal cancer/locally recurrent rectal cancer	10/15
Preoperative (chemo)radiotherapy	
No/yes	11/14
Surgical procedure	
Open/laparoscopic/robotic	0/20/5
Concomitant sacral bone resection	
No/yes	24/1
Operative time, min, median (IQR)	765 (708–851)
Blood loss, mL, median (IQR)	120 (100–290)

IQR: interquartile range.

### 2.2 Surgical Procedures

**FIGURE 1:**
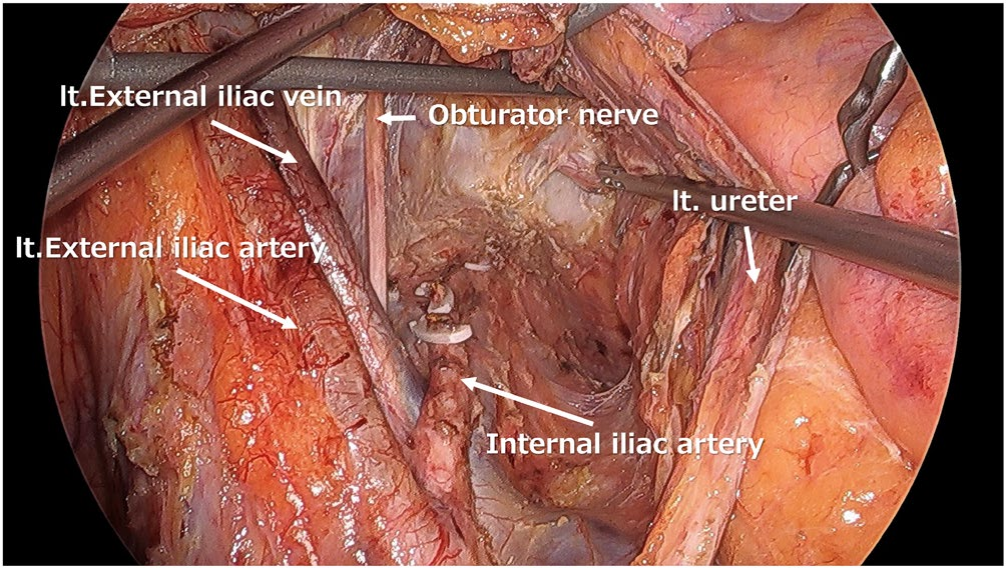
Surgical view after completion of LLND with combined resection of the umbilical artery, obturator vessels, and superior and inferior vesical vessels.

**FIGURE 2:**
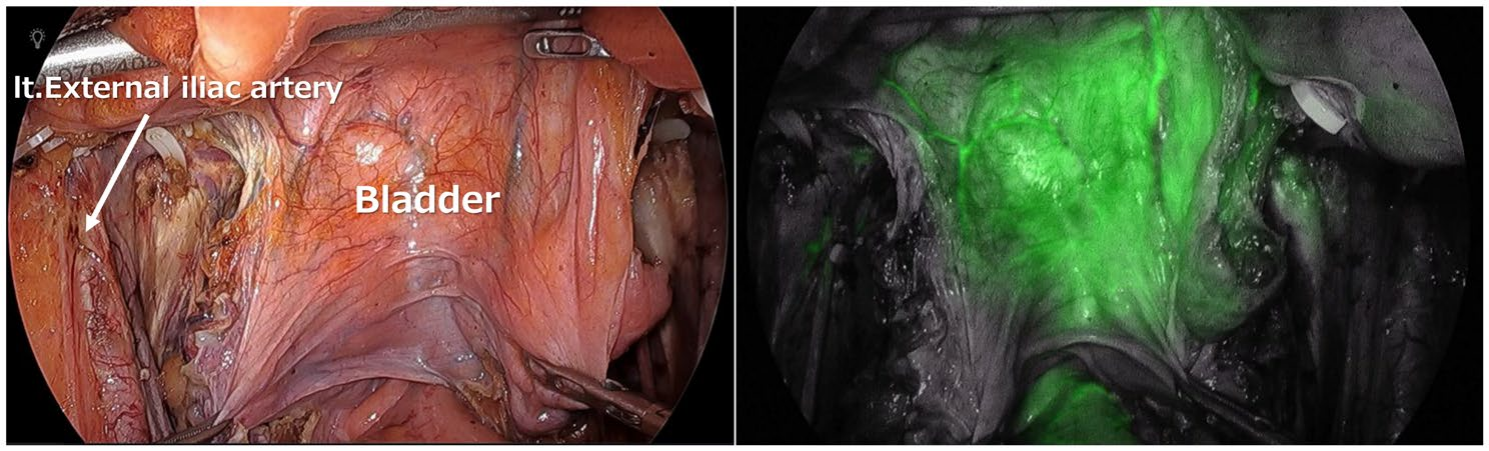
Confirmation of bladder perfusion using ICG after completion of bilateral LLND, distal ligation of both umbilical arteries, and division of the vas deferens (in men) or the round ligament of the uterus (in women).

**FIGURE 3:**
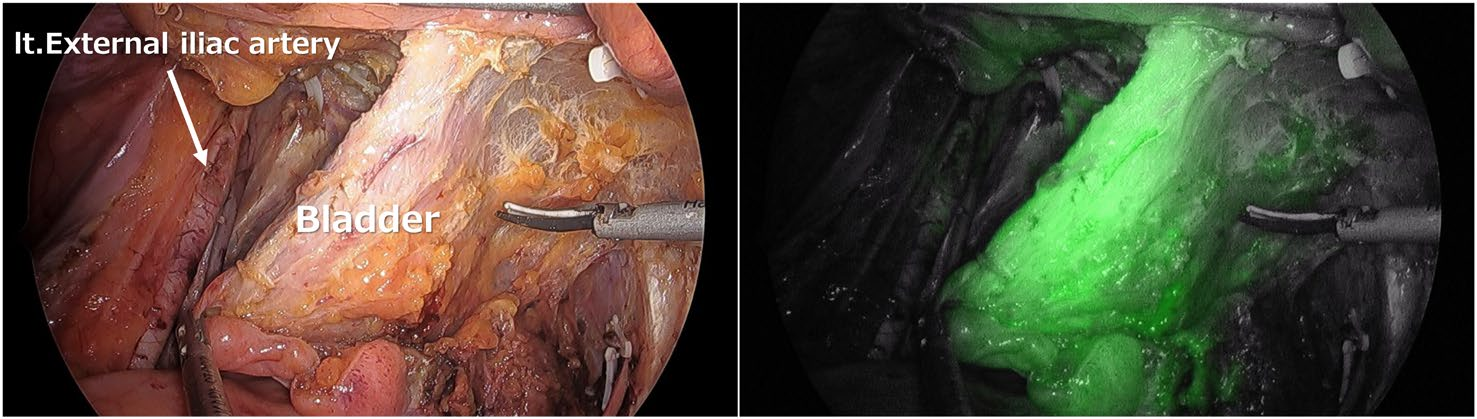
Confirmation of ICG perfusion throughout the bladder wall after bladder mobilization.

The study included patients who underwent TPE with bilateral LLND. During LLND, all ventral branches from the internal iliac vessels—the umbilical vessels, obturator vessels, and superior and inferior vesical vessels—were divided (Figure 1). At that stage, additional division of the distal umbilical arteries and the vas deferens in men or the round ligaments of the uterus in women was also performed, and bladder perfusion was evaluated (Figure 2). To confirm that ICG fluorescence enhanced not only the serosal surface but also the entire bladder wall, a second assessment was conducted after bladder mobilization (Figure 3).

### 2.3 Injection of ICG and Observation of Bladder Perfusion Using Near‐Infrared Fluorescence Imaging

A stock solution of ICG (Diagnogreen; Daiichi Sankyo, Tokyo, Japan) was prepared by dissolving 25 mg of powdered ICG in 10 mL of sterile water. A total of 5.0 mL of the ICG solution was intravenously administered under laparoscopic guidance. In laparoscopic cases, observation of bladder perfusion using near‐infrared fluorescence imaging was performed with either the Stryker 1588 or 1688 Advanced Imaging Modalities (AIM) platform (Stryker Endoscopy, Kalamazoo, MI, USA). Both systems consist of an IRF‐enabled LED light source, light cable, camera control unit (CCU), camera head coupler, and a 30° 10‐mm laparoscope. In robotic cases, the da Vinci Xi HD surgical system (Intuitive Surgical Inc., Sunnyvale, CA, USA) equipped with a near‐infrared laser system was used to assess bladder perfusion.

### 2.4 Statistical Analysis

Numerical data are presented as medians with interquartile ranges (IQRs). Differences between the variables were compared using Fisher's exact test. Differences in quantitative parameters were compared using the Wilcoxon rank‐sum test (Mann–Whitney U test). A *p*‐value < 0.05 was considered statistically significant. All statistical analyses were carried out using JMP Pro software version 17.0.0 (SAS Institute Inc., Cary, NC, USA).

## 3 Results

A total of 19 male and 6 female patients were enrolled, with a median age of 70 years (interquartile range [IQR], 61–72 years). Patient characteristics, including ASA (American Society of Anesthesiologists) physical status classification, presence of diabetes, smoking history, body mass index, primary or locally recurrent rectal cancer, receipt of preoperative (chemo)radiation therapy, surgical approach, concomitant sacrectomy, operative time, and intraoperative blood loss, are summarized in Table 1. Bladder perfusion was successfully visualized in all 25 patients without any complications.

**FIGURE 4:**
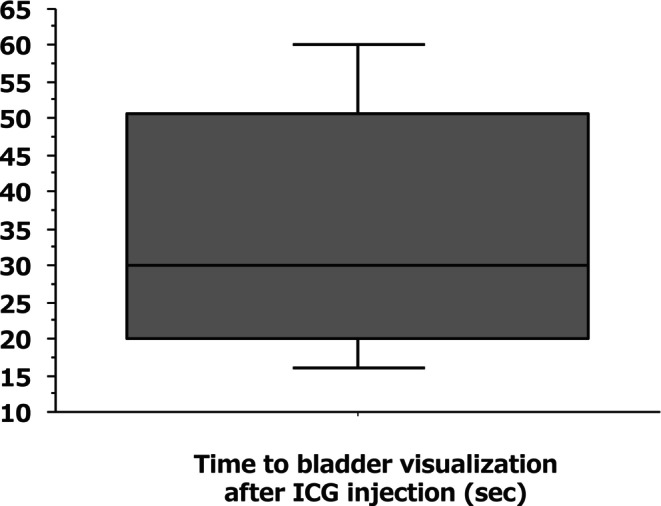
Distribution of time to bladder visualization after ICG injection.

As described above, after completion of bilateral LLND and division of the distal umbilical arteries as well as the vas deferens in males or the round ligament in females, ICG fluorescence imaging was performed to visualize bladder perfusion, and the time to identification of bladder perfusion was measured. In all cases, bladder perfusion was confirmed solely via inflow from the pubic side (Figure 2), with a median time of 30 s (IQR, 20–50 s) (Figure 4). Furthermore, to confirm that perfusion was present throughout the entire bladder wall rather than only in the peritoneum and subperitoneal tissue covering the bladder surface, the bladder was mobilized from the pubic side, and ICG fluorescence imaging was repeated. Fluorescence of the entire bladder wall was confirmed in all cases (Figure 3).

Patients were divided into two groups according to the time required for visualization of bladder perfusion: those with a confirmation time of ≤ 30 s (early visualization group) and those with a confirmation time of > 30 s (delayed visualization group). Patients in the early visualization group were younger, whereas those in the delayed visualization group were significantly older. No associations were found between visualization time and other patient‐ or surgery‐related factors (Table 2).

**TABLE 2:** Comparison of patient characteristics between early and delayed bladder visualization groups.

Variable	Early visualization (≤ 30 s)	Delayed visualization (> 30 s)	*p*
Age	62 (54.5–71)	71.5 (64.78–74)	0.044
Sex (male/female)	9/4	10/2	0.645
Diabetes mellitus (no/yes)	11/2	11/1	1.000
Smoking status (nonsmoker/ex‐smoker, current smoker)	7/6	6/6	1.000
Diagnosis (primary rectal cancer/locally recurrent rectal cancer)	9/4	6/6	0.428
Preoperative (chemo)radiotherapy (no/yes)	6/7	5/7	1.000
Surgical procedure (laparoscopic/robotic)	11/2	9/3	0.645
Operative time (min)	809 (703–889)	757 (703–915)	0.892
Blood loss (mL)	130 (70–485)	110 (100–231)	0.848

*Note:* Values are presented as median (interquartile range, IQR) or number of patients.

## 4 Discussion

In LLND for locally advanced or recurrent rectal cancer, concomitant resection of the internal iliac vessels is often required [3]. The internal iliac artery is a major route of arterial supply to the bladder [10], and it is clinically important to determine whether bladder perfusion can be maintained when several branches to the bladder are divided. However, no previous studies have clarified the extent to which bladder perfusion depends on the internal iliac vessel system.

To accurately evaluate blood supply from non–internal iliac sources, such as branches arising from the pubic side, it is necessary to completely divide and ligate the arterial supply from both internal iliac vessels. Such an evaluation is feasible only in cases undergoing cystectomy, and no previous reports have addressed this issue.

In this study, we demonstrated that bladder perfusion was maintained even after complete interruption of arterial supply from both internal iliac arteries, owing to blood supply flow from the pubic side. Although no major named arteries are considered to directly supply the bladder from the pubic side, perfusion in this region may be maintained by small anastomotic vessels around the pubic symphysis, mainly derived from the inferior epigastric artery [11, 12], and possibly involving fine branches of the accessory pudendal artery when present [13] [14]. Notably, delayed visualization of bladder perfusion was significantly associated with older age. This finding suggests that blood supply from the pubic side may be less efficient in elderly patients, and greater caution may therefore be required when performing extended LLND with internal iliac vessel resection in this population. Therefore, in surgery for locally advanced or recurrent rectal cancer, if partial preservation of internal iliac branches is possible, bladder perfusion may be maintained in combination with blood supply from the pubic side. The limitations of this study include the fact that all cases involved cystectomy; thus, it remains unclear whether ischemia would occur in clinical situations where the bladder is preserved. In addition, the functional outcome of a preserved bladder after such procedures remains uncertain. Furthermore, this study could not precisely determine the extent of internal iliac vessel preservation required to avoid clinically relevant ischemia.

In conclusion, this study is the first to demonstrate that bladder perfusion can be maintained even after complete interruption of both arterial supply and venous drainage from the internal iliac vessels. These findings provide supportive evidence for the safety of concomitant resection of the internal iliac vessels during extended LLND in surgery for locally advanced or recurrent rectal cancer.

## Author Contributions

**Mamoru Uemura:** conceptualization, investigation, methodology, validation, formal analysis, data curation, writing – review and editing, writing – original draft, visualization, supervision, project administration, resources. **Chikako Kusunoki:** writing – review and editing, data curation. **Mao Osaki:** writing – review and editing, supervision. **Hiroshi Kusafuka:** data curation, writing – review and editing. **Satoshi Higuchi:** writing – review and editing, data curation. **Yuki Sekido:** data curation, writing – review and editing. **Mitsunobu Takeda:** data curation, writing – review and editing. **Tsuyoshi Hata:** writing – review and editing. **Atsushi Hamabe:** data curation, writing – review and editing. **Takayuki Ogino:** writing – review and editing, data curation. **Norikatsu Miyoshi:** writing – review and editing. **Koji Munakata:** data curation. **Hirofumi Ota:** data curation. **Yuichiro Doki:** writing – review and editing, supervision. **Hidetoshi Eguchi:** supervision, writing – review and editing.

## Funding

The authors have nothing to report.

## Ethics Statement

This study was approved by the Ethics Committee of Osaka University (16379–3) and was conducted in accordance with the Declaration of Helsinki.

## Consent

Written informed consent was obtained from all participants.

## Conflicts of Interest

The authors declare no conflicts of interest, except that Yuichiro Doki serves as a member of the Annals of Gastroenterological Surgery Editorial Board.
